# Dissolved organic carbon leaching from plastics stimulates microbial activity in the ocean

**DOI:** 10.1038/s41467-018-03798-5

**Published:** 2018-04-12

**Authors:** Cristina Romera-Castillo, Maria Pinto, Teresa M. Langer, Xosé Antón Álvarez-Salgado, Gerhard J. Herndl

**Affiliations:** 1 Department of Limnology and Bio-Oceanography, Center of Functional Ecology, University of Vienna, Althanstraße 14, 1090 Vienna, Austria; 2 CSIC Institut de Ciències del Mar, Passeig Martítim de la Barceloneta 37-49, 08003 Barcelona, Spain; 3 CSIC Instituto de Investigacións Mariñas, Eduardo Cabello 6, 36208 Vigo, Spain; 4 Department of Marine Microbiology and Biogeochemistry, Royal Netherlands Institute for Sea Research, Utrecht University, PO Box 59, 1790 AB Den Burg, The Netherlands

## Abstract

Approximately 5.25 trillion plastic pieces are floating at the sea surface. The impact of plastic pollution on the lowest trophic levels of the food web, however, remains unknown. Here we show that plastics release dissolved organic carbon (DOC) into the ambient seawater stimulating the activity of heterotrophic microbes. Our estimates indicate that globally up to 23,600 metric tons of DOC are leaching from marine plastics annually. About 60% of it is available to microbial utilization in less than 5 days. If exposed to solar radiation, however, this DOC becomes less labile. Thus, plastic pollution of marine surface waters likely alters the composition and activity of the base of the marine food webs. It is predicted that plastic waste entering the ocean will increase by a factor of ten within the next decade, resulting in an increase in plastic-derived DOC that might have unaccounted consequences for marine microbes and for the ocean system.

## Abstract (web summary)

The impact of plastic debris floating at the sea surface on the lowest trophic levels of the food web remains unknown. Here, using leaching experiments, the authors show that plastics release dissolved organic carbon into the ambient seawater that is rapidly taken up by marine microbes stimulating their growth.

## Introduction

Plastic debris represents a contemporary environmental problem affecting marine fauna from small copepods to large mammals^1^. Some animals mistake plastic for food^2^ and, among other impacts, the ingestion of plastic might cause stress, false satiation, reduced growth rates and can affect reproduction^3^. However, the impact of plastics on the lower levels of the food web, dominated by marine microbes, remains largely unknown. Plastic can undergo natural abiotic degradation processes of chemical and mechanical nature, collectively called weathering, leading to its fragmentation. Moreover, biodegradation processes by microbes might also contribute to the degradation of plastics^1^. Plastic litter on beaches and floating in seawater is exposed to solar radiation resulting in the formation of surface cracks and fragmentation into progressively smaller particles, ultimately reaching microscopic sizes^1, 4^. Around 35,000 metric tons (MT) of all the plastics floating in the ocean are smaller than 5 mm (microplastics)^5^. Microplastics can also be directly derived from personal care and cosmetic products or textile fibers entering the marine environment via wastewater discharge^6^. To increase their performance and durability, commercial plastic frequently contains additives. In contact with water, these additives can leach from the plastic into the surrounding water^7^. In this paper, the term leachate is used for any compound (additive or organic substance derived from polymer degradation including any nano-particle that might break off from the plastic surfaces) released from the plastic into the seawater. It has been shown that photo-degradation of plastic can release submicron particles into the aquatic media^8^. With decreasing particle size, the surface to volume ratio of the particle increases, potentially increasing also the concentration of leachates in the surrounding water.

The oceanic DOC pool is one of the largest reduced carbon pools on Earth (662 Pg C)^9^ and it is of similar size as the atmospheric CO_2_ (828 Pg C)^10^. Oceanic DOC is mainly derived from phytoplankton and forms the basic substrate for the microbial food web fueling micro-heterotrophic growth^9, 11^. In this study, we hypothesize that the estimated 250,000 MT of plastics currently floating in the ocean^12, 13^ also contribute to the oceanic DOC pool via leaching. If bioavailable these plastic-leachates might stimulate microbial carbon flux in the ocean, especially in the surface layer. Here we use the term bioavailable DOC for the DOC which is used by heterotrophic bacteria within days. In this regard, it is expected that particles of synthetic polymers are not bioavailable in the timescale of the experiment but only the truly dissolved leachate. Most of the marine studies on plastics focus on the distribution of plastic size classes in the surface ocean^1, 3^. The role of plastics releasing DOC into the ambient seawater, their biodegradation and effects on the marine biota, however, remain largely unknown.

In the present study, the potential contribution of DOC leaching from microplastics to the surface oceanic DOC pool is estimated and the bioavailability of the plastic-derived DOC determined. We calculate that up to 23,600 MT of DOC is annually released by marine plastic litter. In highly contaminated areas, where plastics can be found in concentrations up to 2500 g km^−2^, the leached DOC could make up to 10% of the DOC in the surface microlayer (top 40 µm of the water column). About 60% of the DOC leached from plastics is bioavailable in less than 5 days. However, if exposed to solar radiation, this plastic-derived DOC becomes less labile. Plastic leaching likely gives rise to local hot spots of DOC that can be rapidly remineralized by marine microbes. However, we do not know the fate of the 40% that is not taken up rapidly by bacteria. Taken together, we conclude that increasing plastic waste entering the oceans will have consequences for the marine microbial food web and for the carbon cycling.

## Results

### DOC leaching from plastics

Experiments were performed using commercially available low- and high-density polyethylene (LDPE and HDPE) as well as polypropylene (PP) and polyethylene (PE) from supermarket packaging (see Methods section). PP and PE were used because they represent the most abundant plastics found in the ocean^5, 14^. The plastics were added to autoclaved artificial seawater (ASW) and exposed to artificial solar radiation over a period of 6 days and/or 30 days. Dark treatments and controls without plastics were also performed at the same time in all the experiments. DOC was measured before and after the irradiation period from unfiltered samples. Therefore, in this study DOC includes all the organic carbon compounds released by plastic, from truly dissolved substances to any polymeric nano-plastic that might have been also fragmented from the plastic surface. All plastic types used here leached DOC into the ambient water whether they were irradiated or held in the dark. To calculate the total DOC leaching from the plastics, the DOC concentration of the ASW prior to adding the plastics was used as initial concentration (Table 1).

**Table 1:** DOC concentration leached from plastics during the light and dark incubations

Plastic type	Incubation time (d)	Treatment	Total DOC leached (µg cm^−2^)	Instantaneous DOC leached (µg cm^−2^)	Incubation DOC leached (µg cm^−2^)	DOC consumption during microbial incubation (µmol L^−1^)
LDPE	6	Light	5.8 ± 0.51	3.50 ± 0.51	2.3 ± 0.07	74.0 ± 4.39
		Dark	6.02 ± 1.75	2.81 ± 1.80	3.21 ± 0.41	94.6 ± 16.74
HDPE	6	Light	2.41 ± 0.94	2.03 ± 0.94	0.39 ± 0.05	53.7 ± 5.62
		Dark	2.74*	1.56*	1.18 ± 0.5	104.1 ± 1.50
LDPE	30	Light	6.67 ± 0.90	0.67 ± 1.39	6.00 ± 1.06	92.1 ± 3.74
		Dark	8.92 ± 0.29	8.13 ± 0.59	0.79 ± 0.52	150.4 ± 17.62
HDPE	30	Light	6.28 ± 0.96	5.16 ± 0.96	1.12 ± 0.03	136.3 ± 0.54
		Dark	2.79 ± 0.34	2.42 ± 0.45	0.37 ± 0.29	69.6 ± 17.34
PE packaging	30	Light	0.31 ± 0.16	0.09 ± 0.17	0.22 ± 0.02	27.2 ± 1.16
		Dark	0.26 ± 0.20	0.52 ± 0.39	−0.26 ± 0.33	30.9 ± 3.49
PP packaging	30	Light	2.17 ± 0.13	1.10 ± 0.42	1.07 ± 0.40	55.0 ± 1.77
		Dark	1.61 ± 0.15	1.10 ± 0.20	0.51 ± 0.14	52.5 ± 2.25

Instantaneous DOC leached is the DOC released right after the addition of the plastic to the artificial seawater and prior to the exposure to the artificial solar radiation. Total DOC leached: DOC at the end of the irradiation period minus DOC in artificial seawater before the plastic addition; Incubation DOC leached: DOC at the end of the irradiation period minus DOC after the plastic addition and prior incubation. DOC consumption during microbial incubation: DOC at the end of the microbial experiment minus DOC at the initial time of the microbial experiment. * only one sample of the three replicates. *HDPE* high-density polyethylene, *LDPE* low-density polyethylene, *PE* polyethylene, *PP* polypropylene

**Fig. 1:**
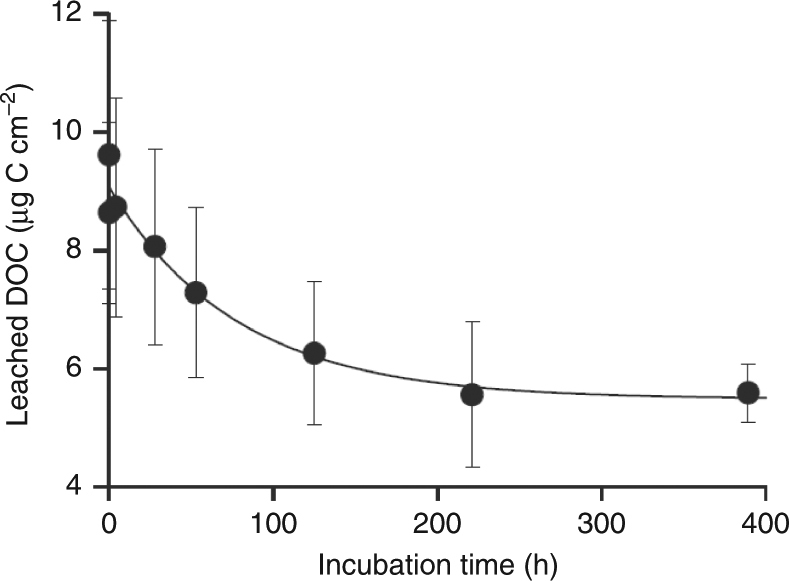
Loss of DOC over time using LDPE. The original DOC concentration of the artificial seawater before plastic addition was subtracted from all the points. Thus, the first point represents the maximum leaching after the plastic addition. Error bars show the standard deviation of the mean of three replicates. First order rate constant = −0.013 h^−1^ ± 0.003; *R*_2_ = 0.97; *p* < 0.001. LDPE low-density polyethylene, DOC dissolved organic carbon

LDPE and HDPE, either after 6 days or after 1 month of sterile incubation, leached between 2.4 and 8.9 μg C cm^−2^ of plastic surface (total DOC leached; Table 1). Plastics used as packing material released less DOC amounting to 0.26–0.31 μg C cm^−2^ and 1.6–2.2 μg C cm^−2^ plastic surface for PE and PP, respectively. A time course experiment conducted in the dark using LDPE added to artificial seawater confirmed that the highest leaching of DOC from plastics occurs initially, when plastics get in contact with seawater (Fig. 1). Thereafter, DOC concentration exponentially decreased (about 45%) until reaching a constant leaching of about 5.5 μg C cm^−2^, roughly similar to the total DOC leached in our irradiation experiments (Table 1). The first-order constant of DOC lost was −0.0135 h^−1^, i.e., half of the missing leached DOC was lost in about 50 h (Fig. 1).

Accordingly, the effect of artificial solar radiation on the leaching of DOC from plastics floating in sterile seawater was also determined (Incubation DOC leached; Table 1). The DOC concentration immediately after adding the plastics was used as initial DOC concentration. The effect of artificial solar radiation varied depending on the plastic material and the duration of exposure. Over a 6-day incubation period under artificial solar radiation, we measured less DOC leached from LDPE and HDPE (2.30 and 0.39 μg C cm^−2^ of plastic surface, respectively) than from their corresponding dark treatments (3.21 and 1.18 μg C cm^−2^, respectively). For HDPE, however, the differences were not significant (*t* test, *p* > 0.05). In contrast, over 1-month incubation period, irradiated LDPE and HDPE released significantly more DOC (*t* test, *p* < 0.05) (6 and 1.12 μg C cm^−2^, respectively) than their corresponding dark treatments (0.79 and 0.37 μg C cm^−2^, respectively). The packaging plastic (PE and PP) did not exhibit significant differences in the DOC leaching between artificial solar radiation and the corresponding dark controls (*t* test, *p* > 0.05).

### Microbial utilization of DOC leached from plastics

As plastics floating at the sea surface release DOC, we aimed at determining the bioavailability of plastic-derived DOC and its influence on the microbial community. Therefore, after removing the plastic pieces from the seawater incubated in the previous experiments, we inoculated this water with a natural bacterial community from surface waters of the Adriatic Sea (nine parts of water with plastic-leachate: one part of inoculum). The samples were incubated in the dark until microbes reached stationary phase.

**Fig. 2:**
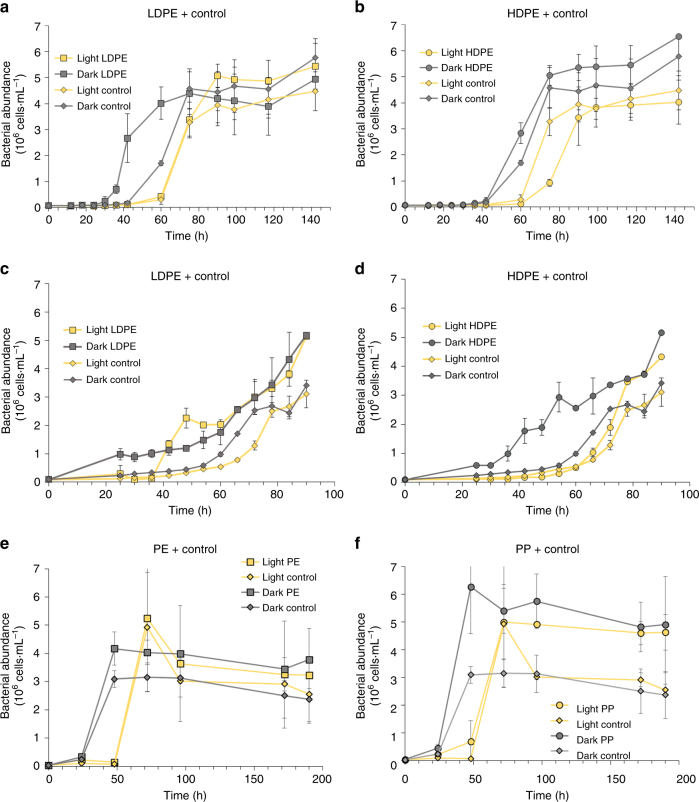
Bacterial abundance during incubation with plastic leachates. Bacterial abundance (cell mL^−^^1^) during the incubation with DOC leached from plastics previously irradiated for 6 days using **a** LDPE and **b** HDPE; for 30 days using **c** LDPE and **d** HDPE; for 30 days using **e** PE and **f** PP. Error bars represent the standard deviation of the mean of the triplicate cultures. HDPE high-density polyethylene, LDPE low-density polyethylene, PE polyethylene, PP polypropylene

**Fig. 3:**
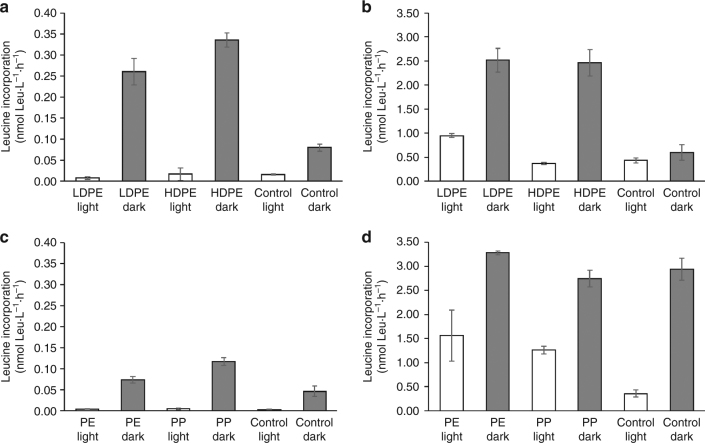
Leucine incorporation in the 30-days plastic leaching experiments. Leucine incorporation (nmol Leu L^−1^ h^−1^) for: LDPE and HDPE treatments after **a** 24 h and **b** 48 h of incubation; PE and PP packaging plastic after **c** 24 h and **d** 48 h of incubation. Error bars represent the standard deviation of the mean of the triplicate cultures. HDPE high-density polyethylene LDPE low-density polyethylene, PE polyethylene, PP polypropylene

In the treatments previously containing plastics (thereafter plastic treatments) and held in the dark, bacteria grew faster than in the previously irradiated plastic treatments or in the controls without plastics (Fig. 2). In general, in the irradiated plastic samples, bacterial abundance reached higher values (Fig. 2c, d) than in the irradiated control without plastic but in some cases, the differences were low or not significant (*t* test, *p* < 0.05; Fig. 2a, b, e, f). The higher bacterial abundance in the plastic treatments held in the dark regarding the irradiated and no-plastic treatments was also consistent with higher leucine incorporation rates (a proxy for biomass production) (Fig. 3). After 24 h of incubation, leucine incorporation in LDPE and HDPE held in the dark (0.26 nmol Leu L^−1^ h^−1^ and 0.34 nmol Leu L^−1^ h^−1^, respectively) was at least one order of magnitude higher (*t* test, *p* < 0.05) than in the irradiated treatments (0.75 × 10^−2^ and 1.67 × 10^−2^ nmol Leu L^−1^ h^−1^, Fig. 3a). This trend was also observed after 48 h of incubation (Fig. 3b).

At the end of the bacterial incubation experiments, there were no significant differences (*p* *<* 0.05) in bacterial abundance between plastic treatments and the controls without plastics (Fig. 2). Also, there was no clear pattern in the maximum bacterial abundance reached among the different types of plastics. All the treatments reached a maximum bacterial abundance of about 6×10^6^ cells mL^−1^. Bacteria started to grow slightly earlier in the LDPE and packaging PE and PP than in the HDPE treatments (Fig. 2). After 24 h of incubation, bacterial leucine incorporation was significantly higher (*p* < 0.05) in the plastic treatments than in the non-plastic treatments, especially under dark conditions. When packaging plastic was used, leucine incorporation rates measured after 24 h of incubation were lower than in the other plastic types. Nevertheless, leucine incorporation in the dark plastic treatments (7.41 × 10^−2^ and 0.12 × 10^−2^ nmol Leu L^−1^ h^−1^ for PE and PP, respectively) was higher than in the corresponding irradiated (4.25 × 10^−3^ and 4.97 × 10^−3^ nmol Leu L^−1^ h^−1^ for PE and PP, respectively) treatments (Fig. 3c). After 48 h of incubation, bacterial leucine incorporation in the dark treatments was significantly higher (*p* < 0.05) than in the irradiated treatments. However, the dark control was not significantly different from the dark treatments containing plastics (Fig. 3d).

The bacterial growth efficiency (BGE, as %) was calculated from the increase in bacterial carbon biomass and the concomitant decrease in DOC in the incubations over time. An average BGE of 12% ± 10% was obtained for plastics (irradiated and dark treatments) and 19% ± 8% for the control treatments (irradiated and dark) without plastics. No significant differences were found in the BGE between treatment with and without plastic neither for irradiated and dark treatments. These BGE values are comparable with those of coastal waters^15^.

**Fig. 4:**
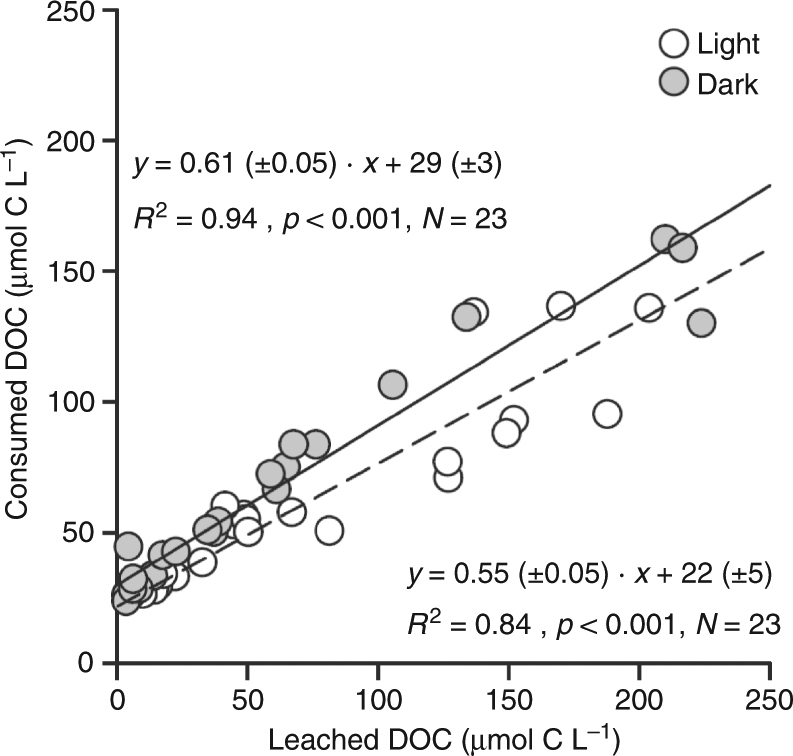
Relation between DOC consumption by microbes and DOC leaching from plastics. DOC in µmol C L^−^^1^. Linear regression equation (Model II) using all data: *Y* = 0.58 (±0.03)⋅*X* + 45.43 (±3.03) (*R*^2^ = 0.87; *p* *<* 0.001; *N* = 46). Open circles: irradiated samples; Gray circles: non-irradiated samples. DOC dissolved organic carbon

A strong positive linear relationship between DOC leaching and bacterial DOC consumption (*R*^2^ = 0.87; *p* < 0.001; *N* = 46, all plastic experiments included, Model II regression^16^, Fig. 4) indicated that DOC leaching from plastics, either irradiated or held in the dark, is utilized by marine microbes. The regression slope indicates that 58% ± 3% of the leached DOC is taken up by microbes at the time scale of the incubations.

## Discussion

All plastic types used here leached DOC into seawater with a major fraction being released in the initial phase when plastics first have contact with seawater (60% ± 26%; see Instantaneous DOC leached in Table 1). About 45% of the leached DOC is progressively lost during the first 200 h as shown in the kinetic experiment conducted in the dark (Fig. 1). Since in this experiment photo-oxidation cannot be the cause of the DOC decline, the loss of DOC could be due to sorption onto the plastic, a loss of volatile leachates or a combination of both. The sorption–desorption capacity of plastics is well documented^17^; thus a part of the DOC might re-adsorb onto plastics until an equilibrium is reached. The sorption of organic compounds onto plastic can be a fast process. Bakir et al.^18^ found that some organic pollutants absorbed onto polyethylene floating in seawater reaching an equilibrium within 24–48 h. However, other studies determined a longer period (>20 days) to reach equilibration for organic pollutants absorbing onto polyethylene and polypropylene^17^. In our study, the equilibrium was reached after about 200 h. That could include the sorption of the DOC that was already present in seawater. However, in our study, this amounted to only 5% of the total DOC before it started to decrease. So, if some sorption of the DOC from seawater happened in our experiments, this was minimal compared to the DOC leachate. In nature, the sorption of natural DOC onto plastics could compete with microbial DOC uptake disrupting the natural lower trophic level processes. Another explanation for the observed loss of DOC might be the production of volatile organic compounds resulting from degradation of plastics^19^. The strong plastic specific smell at the end of our incubation experiments (both in the irradiated and non-irradiated) suggests that some of the leached DOC is volatile. In irradiated samples, in addition to the possible direct release of organic volatiles from the plastics, solar radiation might have caused photo-degradation of the released non-volatile compounds resulting in CO, CO_2_, or organic volatiles not measurable with our method to determine DOC. Possible photo-degradation products from plastic are propane, propene, ethane, ethylene, butane, and hexene^20^ among others. The method for measuring DOC applied in this study only accounts for the non-volatile fraction because the seawater samples are sparged with N_2_ gas prior to the analysis to remove all the inorganic carbon which, however, also results in the loss of the volatile DOC fraction (see Methods section). Thus, it is likely that plastics exposed to solar radiation leach more DOC than is measured with the approach used here.

It has been estimated that between 4.8 × 10^12^ and 12.7 × 10^12^ MT of plastics entered the ocean in the year of 2010^21^. Assuming that floating plastics have an average density of 0.96 g cm^−3^ (see Methods section) and given our range of DOC leaching (0.26–8.92 µg C cm^−2^; Table 1), we estimate that between 260 and 23,600 MT of DOC year^−1^ leach from those plastics. Previous estimates indicate that 5.25 × 10^12^ pieces of plastic particles weighing 268,940 MT are currently floating at the sea surface^5, 12^, i.e., much less than the plastic entering the ocean annually^21^. This indicates that the majority of the plastics entering the ocean is not floating at the surface. Missing plastic likely sunk in the water column and is buried in marine sediments or it is too small to be caught by the commonly used nets to collect marine plastics (about 200 µm)^5^. In the subtropical gyres, plastic concentrations can be as high as 2500 g km^−2^ ^5, 12^. By extrapolating our results to the concentration of plastics in the ocean, we estimate that 2500 g km^−2^ of plastics would leach between 0.14 and 4.65 g DOC km^−2^. Considering a 40 µm thick surface microlayer in which these plastics are floating and leaching, 2500 g km^−2^ of plastics leach between 0.28 and 9.68 µmol C L^−1^ within 5 days. DOC concentrations of about 110 µmol L^−1^ have been measured in the surface microlayer of the oligotrophic gyre of the North Atlantic^22^. Thus, up to 10 ± 0.3% of the DOC in the surface microlayer might originate from plastics. This could create local hot spots of high DOC concentrations stimulating microbial activity.

Even if the plastic types used in our experiments are the most abundant plastics found in the ocean (polyethylene and polypropylene)^14^, there is a high diversity of plastic polymers in the ocean. Other plastic types might release different amounts of DOC than those examined here. Therefore, the values presented here are only tentative estimates. Aging and degradation of plastic causes its breakdown into small pieces^23^, a mechanism, which could potentially increase DOC leaching due to the increase in surface area relative to its volume. The plastics used in our experiments did not show any signs of aging to the naked eye after the exposure to artificial solar radiation. Moreover, since the missing plastic mentioned above is not taken into account here, our estimates of DOC leaching from plastics are likely very conservative.

Plastics could also contribute to the DOC measurements through their direct presence in the samples. Nanoplastics (<100 nm) are either derived from fragmentation of larger particles or directly from products for personal care, cosmetics, or textile fibers^6, 24, 25^. A recent study showed that plastic particles smaller than 2 µm are generated upon exposure of polystyrene to solar radiation^8^. A high number of these polymer particles were found to be smaller than 800 nm ^8^. In our experiments, any nanoparticle formed would be included in the DOC pool since we did not filter the sample before and after the irradiation experiment. However, we did not find significant differences in DOC leaching between irradiated and dark samples in most of the experiment, indicating that sub-micrometer particles from photo-degradation were not generated to a significant extent in our experiments. DOC is operationally defined as the fraction of organic matter passing through a 0.2–0.7 µm filter^26^. Therefore, any particle smaller than 700 nm would pass the most commonly used filters for DOC analysis (Whatman GF/F, 0.7 µm pore size) and consequently, will contribute to the measured DOC. Thus, special care should be taken when collecting DOC samples in plastic contaminated areas.

Leaching of DOC from plastics influences the microbial activity and carbon cycling in the ocean. The bioavailable fraction of the leached DOC was (insignificantly) higher when the plastic was kept in the dark than under artificial solar radiation (61% ± 3% in the dark vs. 55% ± 5% in the light treatments). Even in the treatments with only low levels of DOC leaching (e.g., PE packaging plastic), dark conditions stimulated the response of the bacterial community, as indicated by the DOC utilization and by an earlier increase in microbial abundance in the plastic incubations than in the controls without plastic. Even where no differences in the DOC leaching between the light and dark treatments were observed, microbial growth started earlier in the dark treatments containing plastics indicating that irradiated DOC derived from plastics might undergo photochemical transformation affecting microbial growth. Photodegradation of plastics is known to produce free radicals^20^ potentially inhibiting bacterial growth^27^. Therefore, the lower bacterial abundance and leucine incorporation in the treatments exposed to artificial solar radiation might be due to the formation of microbial inhibitors. The large amount of plastics potentially present in the deeper water layers or in sediments^5^ is not exposed to solar radiation. Thus, plastic-derived DOC could also potentially enhance microbial growth in layers well below the oceanic surface layer. Our leaching experiments were performed under abiotic conditions. In the ocean, however, microbes will have access to the plastic-derived DOC immediately upon its release, resulting in ~45% more DOC, potentially available, than the estimates given in Table 1 (Total leached DOC). Thus, re-sorption of leached DOC onto the plastics would be largely prevented if microbes would efficiently take up the DOC as soon as it is released from the plastics.

DOC leaching from plastics and its effect on microbial activity might be important in areas with high plastic concentrations (e.g., subtropical gyres or near shore waters), especially in the surface microlayer. The quantity of plastic waste entering the oceans is predicted to increase by up to one order of magnitude by the year 2025 (20) resulting in plastic-derived DOC of up to 236,000 MT per year in the global ocean, with potential major consequences for marine microbes and the carbon cycling in the oceanic system.

## Methods

### Plastic leaching experiments

A total of six experiments were performed using four different types of plastics (low density polyethylene (LDPE), high density polyethylene (HDPE), polyethylene (PE), and polypropylene (PP)) and two exposure times with artificial solar radiation, 6 days and 30 days. An additional experiment to study the kinetics of DOC leaching from plastics was performed in the dark using LDPE.

Artificial photosynthetic active radiation (PAR) was provided by a HQI-T Powerstar lamp (250 W, Osram), UV-A radiation by 2 Philips TL100W/10 R fluorescent tubes, and UV-B radiation by 2 UVA-340 fluorescent lamps (Q-Panel Company, UK). The radiation intensity for each wavelength or wavelength range was as follows: PAR (400–700 nm), 700 µmol m^−2^ s^−1^; 380 nm, 28.47 µW cm^−2^ nm^−1^; 340 nm, 16.31 µW cm^−2^ nm^−1^; 320 nm, 7.95 µW cm^−2^ nm^−1^; 305 nm, 1.09 µW cm^−2^ nm^−1^. The radiation dose rate represents the solar radiation in the subtropical North Atlantic Gyre measured at noon at 15 m depth^28^. Artificial solar radiation was measured at 305, 320, 340, 380 nm, and PAR with a Biospherical PUV-510 radiometer using a correction factor for the 305 nm channel as suggested by Kirk^29^. The light treatments received continuous artificial solar radiation.

Polyethylene plastic (LDPE and HDPE) was obtained from GoodFellow as plastic film (LDPE, 0.5 mm thickness) and pellets (HDPE, ~4 mm diameter). PE from fruit bags and PP from fruit packaging, both translucent, were obtained from a local supermarket in Vienna (Austria). LDPE, PE, and PP were cut into ~7 mm squares. Plastic pieces (63 squares for LDPE, PE and PP, and 123 pellets for HDPE experiments) were added to 250 mL of sterilized (autoclaved at 121 °C for 20 min) artificial seawater in quartz tubes for the treatments receiving artificial solar radiation. Dark controls were established in the same way but using borosilicate bottles wrapped in aluminum foil. Light and dark controls without plastics were also performed in the same way as the samples with plastics. All the treatments and controls were performed in triplicate. Light and dark samples were placed in the solar simulator under the radiation conditions described above during 6 days (LDPE and HDPE) and 30 days (LDPE, HDPE, PE and PP). A flow-through water bath maintained the temperature of the incubation flasks at 23 °C.

DOC was measured from the unfiltered artificial seawater before and after the incubation period to estimate the DOC leaching from the plastic material and the effect of the radiation on it. DOC released was normalized to the surface area of the whole plastic used in every flask. Bacterial abundance was measured at the end of the experiment to check whether bacterial growth took place in the plastic leaching experiments. However, bacterial abundance in these experiments was always below 10^4^ mL^−1^.

### Bioavailability of DOC leaching from plastics

Following the exposure to artificial solar radiation, plastic pieces were removed from the incubation flasks and 0.8 µm filtered surface seawater from the North Adriatic Sea was added to the incubation water at a ratio of 9:1 (incubation water: 0.8 µm filtered). The treatments were amended with NH_4_Cl and NaH_2_PO_4_ to a final concentration of 10 and 2 µm L^−1^, respectively, to avoid growth limitation by either nitrogen or phosphorus availability. The flasks were incubated in the dark at 23 °C until the mixed microbial community reached stationary phase. Samples for microbial abundance were collected every 6–12 h for the LDPE and HDPE experiments and daily for the PE and PP experiments. DOC samples were collected at the beginning of the incubation and at the end of the exponential growth phase of the microbial community. At the end of this incubation experiment, DOC samples were filtered through combusted Whatman GF/F filters to remove bacteria. In the 30 days experiments with different types of plastics (LDPE, HDPE, PE, and PP), heterotrophic microbial production was measured 24 and 48 h after inoculating the microbial community as described below.

An additional experiment was performed to study the kinetics of the DOC leaching from plastics in which 63 LDPE squares were added to 300 mL of artificial seawater. The samples were kept in the dark at 23 °C and DOC samples were collected at different times over a 2-weeks period, with a higher frequency in the initial phase of the kinetic study.

### DOC and microbial analysis

DOC samples were collected unfiltered for the initial and final time of the irradiation experiment as well as the kinetic experiment. However, they were filtered at the end of the microbial incubation experiment (Whatman GF/F filters pre-combusted at 450 °C for 4 h) to remove bacteria. Water for DOC analyses was collected in pre-combusted 20 mL glass vials, acidified with concentrated HCl to pH < 2 and stored at 4 °C until analysis. DOC was measured with a Shimadzu TOC-V organic carbon analyzer^30^ after removal of CO_2_ by vigorous sparging with high purity N_2_. Therefore, only the non-volatile fraction of DOC is measured. The accuracy was tested daily with the DOC reference materials provided by D.A. Hansell (University of Miami). We obtained average concentrations of 43.3 ± 0.3 μmol L^−1^ for the deep ocean reference (Batch 16—2016) minus blank reference materials. The nominal DOC value provided by the reference laboratory was 43–45 μmol L^−1^.

The global budget of DOC leaching from plastic in the ocean was calculated assuming a plastic density of 0.96 g cm^−3^, a plastic thickness of 0.1 mm and the two sides of the plastic film.

Bacterial abundance was measured collecting aliquots of 1.5–2 mL from each bottle, fixed with 0.5% glutaraldehyde (final concentration, Sigma-Aldrich), stored in the dark for 10 min and subsequently frozen at −80 °C until further processing. Bacterial abundance was measured by flow cytometry (BD FACSAria IIu) following the method described elsewhere^31^.

Leucine incorporation rates of the microbial communities were measured by adding 20 nmol L^−1^ [^3^H]-leucine (final concentration, specific activity 120 Ci mmol L^−1^) to triplicate 1.5 mL samples. Duplicate TCA (trichloroacetic acid)-killed blanks (5% final concentration) were treated in the same way as the samples^32^. Samples and blanks were incubated in the dark at 20 °C for 2 h. Incubations were terminated by adding TCA (5% final concentration) to the samples. Bacterial proteins were precipitated by two successive centrifugation steps (12,000 × *g* for 10 min), including a washing step with 1 mL of 5% TCA. The samples were air-dried before adding 1 mL of liquid scintillation cocktail. After 24 h, the radioactivity was determined in a scintillation counter (Packard Tri-Carb 1600TR). The disintegrations per minute (DPMs) of the blanks were subtracted from the mean DPMs of the respective samples and the resulting DPMs converted into leucine incorporation rates.

### Data availability

The authors declare that the data supporting the findings of this study are available within the article.

## References

[1] Andrady AL (2011). Microplastics in the marine environment. Mar. Pollut. Bull..

[2] Savoca MS, Wohlfeil ME, Ebeler SE, Nevitt GA (2016). Marine plastic debris emits a keystone infochemical for olfactory foraging seabirds. Sci. Adv..

[3] Auta HS, Emenike CU, Fauziah SH (2017). Distribution and importance of microplastics in the marine environment: A review of the sources, fate, effects, and potential solutions. Environ. Int..

[4] Kalogerakis N (2017). Microplastics generation: onset of fragmentation of polyethylene films in marine environment mesocosms. Front. Mar. Sci..

[5] Cózar A (2014). Plastic debris in the open ocean. PNAS.

[6] Fendall LS, Sewell MA (2009). Contributing to marine pollution by washing your face: microplastics in facial cleansers. Mar. Pollut. Bull..

[7] Suhrhoff TJ, Scholz-Böttcher BM (2016). Qualitative impact of salinity, UV radiation and turbulence on leaching of organic plastic additives from four common plastics—a lab experiment. Mar. Pollut. Bull..

[8] Lambert S, Wagner M (2016). Characterisation of nanoplastics during the degradation of polystyrene. Chemosphere.

[9] Hansell DA, Carlson CA, Repeta DJ, Reiner S (2009). Dissolved organic matter in the ocean. Oceanography.

[10] Prather MJ, Holmes CD, Hsu J (2012). Reactive greenhouse gas scenarios: systematic exploration of uncertainties and the role of atmospheric chemistry. Geophys. Res. Lett..

[11] Romera-Castillo C, Letscher RT, Hansell DA (2016). New nutrients exert fundamental control on dissolved organic carbon accumulation in the surface Atlantic Ocean. PNAS.

[12] Eriksen M (2014). Plastic pollution in the World’s oceans: more than 5 trillion plastic pieces weighing over 250,000 tons afloat at sea. PLOS One.

[13] van Sebille E (2015). A global inventory of small floating plastic debris. Environ. Res. Lett..

[14] Suaria G (2016). The mediterranean plastic soup: synthetic polymers in Mediterranean surface waters. Sci. Rep..

[15] Del Giorgio PA, Cole JJ (1998). Bacterial growth efficiency in natural aquatic systems. Annu. Rev. Ecol. Evol. Syst..

[16] Sokal F. F. & Rohlf F. J. Introduction to biostatistics (W. H. Freeman & Company, San Francisco, 1984).

[17] Karapanagioti HK, Klontza I (2008). Testing phenanthrene distribution properties of virgin plastic pellets and plastic eroded pellets found on Lesvos island beaches (Greece). Mar. Environ. Res..

[18] Bakir A, Rowland SJ, Thompson RC (2014). Transport of persistent organic pollutants by microplastics in estuarine conditions. Estuar., Coast. Shelf Sci..

[19] Curran K, Strlič M (2015). Polymers and volatiles: using VOC analysis for the conservation of plastic and rubber objects. Stud. Conserv.

[20] Gewert B, Plassmann MM, MacLeod M (2015). Pathways for degradation of plastic polymers floating in the marine environment. ESPI.

[21] Jambeck JR (2015). Plastic waste inputs from land into the ocean. Science.

[22] Reinthaler T, Sintes E, Herndl GJ (2008). Dissolved organic matter and bacterial production and respiration in the sea-surface microlayer of the open Atlantic and the western Mediterranean Sea. Limnol. Oceanogr..

[23] GESAMP. *Sources, fate and effects of microplastic in the marine environment: a global assessment.*Available at: http://ec.europa.eu/environment/marine/good-environmental-status/descriptor-10/pdf/GESAMP_microplastics%20full%20study.pdf (International Maritime Organization, 2015).

[24] Browne MA (2011). Accumulation of microplastic on shorelines woldwide: sources and sinks. Environ. Sci. Technol..

[25] Koelmans, A. A., Besseling, E. & Shim, W. J. *Marine Anthropogenic Litter. *Vol. 12 (eds Bergmann, M., Gutow, L. & Klages, M.) 325–340 (Springer, Berlin, 2015).

[26] Hansell, D. A. & Carlson. C. A. *Biogeochemistry of Dissolved Organic Matter* 2nd edn, Ch. 2 (Academic Press, Oxford, 2015).

[27] Anesio AM, Granéli W, Aiken GR, Kieber DJ, Mopper K (2005). Effect of humic substance photodegradation on bacterial growth and respiration in lake water. Appl. Environ. Microbiol..

[28] Obernosterer I, Ruardij P, Herndl GJ (2001). Spatial and diurnal dynamics of dissolved organic matter (DOM) fluorescence and H_2_O_2_ and the photochemical oxygen demand of surface water DOM across the subtropical Atlantic Ocean. Limnol. Oceanogr..

[29] Kirk JTO (1994). Optics of UV-B radiation in natural waters. Arch. Hydrobiol. Beih..

[30] Álvarez-Salgado XA, Miller AEJ (1998). Simultaneous determination of dissolved organic carbon and total dissolved nitrogen in seawater by high temperature catalytic oxidation: conditions for precise shipboard measurements. Mar. Chem..

[31] Del Giorgio PA, Bird DF, Prairie YT, Planas D (1996). Flow cytometric determination of bacterial abundance in lake plankton with the green nucleic acid stain SYTO 13. Limnol. Oceanogr..

[32] Smith DC, Azam F (1992). A simple, economical method for measuring bacterial protein synthesis rates in seawater using ^3^H-leucine. Mar. Microb. Food Webs.

